# Predictors of Family Recruitment in a Program of Genetic Cascade Screening for Familial Hypercholesterolemia

**DOI:** 10.5935/abc.20180193

**Published:** 2018-10

**Authors:** Maria Cristina de Oliveira Izar, Francisco Antonio Helfenstein Fonseca

**Affiliations:** Disciplina de Cardiologia - Escola Paulista de Medicina - Universidade Federal de São Paulo, São Paulo, SP - Brazil

**Keywords:** Hyperlipoproteinemia Type II/genetic, Lipid Metabolism Disorders, Hyperlipoproteinemia/prevention &control, Family Health Strategy

Familial hypercholesterolemia (FH) is a common inherited disease affecting lipid metabolism; it is associated with lifelong exposure to high levels of LDL-cholesterol, and premature atherosclerotic cardiovascular disease. FH imposes an enormous burden on patients and their relatives, due to years of life lost, and particularly, for not being diagnosed as an entity.^1^

In spite of the high LDL-cholesterol and even after an atherosclerotic event, a large proportion of individuals with FH remains undiagnosed.^2^^,^^3^ Criteria for diagnosing FH are based on clinical findings, family history, LDL-cholesterol levels, and genetic testing (Simon Broome or Dutch Lipid Clinic Network), or on the LDL-cholesterol levels alone (US MED PED).^4^ However, FH phenotypes can vary, and the lack of physical signs (15-30% of patients with genetic diagnosis of FH present xanthomas or corneal arcus, and 5% have xanthelasma) can contribute for the underdiagnosis of FH. ^5^^-^^7^

Genetic testing using a panel that includes FH-causing genes (*LDLR*, *APOB*, *PCSK9*, and *LDLRAP-1*) is the best approach to identify probands.^1^^,^^4^ When cascade screening is proposed to a family with a confirmed genetic case of FH, the costs for this screening program are much lower and are considered a cost-effective intervention, enabling early diagnosis and treatment of the affected relatives. One problem with cascade screening is how to have a high proportion of relatives adhering to the screening program.^8^^-^^11^

Silva-Souza, et al.,^12^ in the article entitled *Predictors of Family Recruitment in a Program of Genetic Cascade Screening for Familial Hypercholesterolemia* identified the best predictors of genetic family screening, using characteristics derived from their probands.^12^ From January 2011 to July 2015, 183 probands (confirmed for FH by genetic testing) had their 1^st^ degree family members recruited for the cascade program. The response variable was the number of relatives that adhered to the recruitment.^13^ Study variables were derived from clinical and socioeconomic characteristics of the index cases. A linear negative binomial regression model was used to test predictors. Reference origin from the *site* of cascade screening vs. tertiary prevention, LDL-cholesterol in the proband, and family history were independent predictors for a higher number of recruited subjects.

There are a number of reasons that would reinforce the need and the importance to adhere to a genetic cascade screening program. The costs are lower than when a proband is diagnosed,^10^ it is a predictor of coronary disease,^14^ adherence to lipid-lowering drugs can be enhanced, and the treatment can be initiated earlier in life.^14^ A structured follow-up of the screened individuals should be performed to assure early and continuous treatment. Most concerns related to lack of adherence to screening are related to patient/relatives education, and physician inertia. Strategies to address these issues and mitigate the burden of atherosclerotic disease in this population should be developed.
